# An Overview of Novel Agents for Cervical Cancer Treatment by Inducing Apoptosis: Emerging Drugs Ongoing Clinical Trials and Preclinical Studies

**DOI:** 10.3389/fmed.2021.682366

**Published:** 2021-07-28

**Authors:** Lei Liu, Min Wang, Xianping Li, Sheng Yin, Bingqi Wang

**Affiliations:** Department of Laboratory Medicine, Second Xiangya Hospital, Central South University, Changsha, China

**Keywords:** novel agents, clinical trials, apoptosis, pre-clinical studies, cervical cancer

## Abstract

As the leading cause of cancer death, cervical cancer ranks fourth for both incidence and mortality. Cervical cancer incidence and mortality rates have reportedly decreased over the last decades thanks to extensive screening and widespread vaccination against human papilloma virus. However, there have been no major improvements concerning platinum-based chemotherapy on the survival of advanced cervical cancer. Thus, novel agents are urgently needed for the improvement of therapeutic effect. With the development of molecular biology and genomics, targeted therapy research has achieved a breakthrough development, including anti-angiogenesis, immune checkpoint inhibitors, and other treatments that are efficient for treatment of cervical cancer. Apoptosis is a crucial process for tumor progression. Drugs directed at inducing tumor-cell apoptosis are regarded as important treatment modalities. Besides, a number of novel compounds synthesized or derived from plants or microorganisms exhibited prominent anti-cancer activity by changing the apoptotic balance in cervical cancer. In this review, we summarized new target therapy drugs ongoing clinical trials that are used for treatment of cervical cancer. Further, we classified novel agents with a focus on improvement of therapeutic effect pre-clinically. To summarize, we also discussed application prospects of the new uses of old drugs and drug combinations, to provide researchers with new ideas for cervical cancer treatment.

## Introduction

Cervical cancer, which is ranked fourth for both incidence and mortality, is the leading cause of morbidity and cancer deaths throughout the world (1). In 2018, there were 8,400,000 new cases of female tumors and 4,200,000 deaths, and cervical cancer accounted for 6.6 and 7.5% of female tumor morbidity and mortality (1). There has been a significant decrease in the incidence and death rate of cervical cancer due to effective Pap smear screening and application of vaccines. However, the prognosis of patients with advanced/recurrent cervical cancer is particularly poor, and their chance of a 1-year relative survival rate is only 10–20% (2). These numbers indicate that there is a need of improvement concerning current treatment strategies, mainly including radiotherapy and chemotherapy. Further, new strategies or therapeutic agents are required to overcome the persistence of human papilloma virus (HPV).

Apoptosis is a spontaneous process in regular cells after physiological or pathological stimuli. Apoptosis is the autophagic cell death that is controlled by genes for maintaining a stable environment. Apoptosis is an active process involving the activation, expression, and regulation of a series of genes. Therefore, modulation of specific molecular pathways leading to apoptosis is a primary strategy for cervical cancer treatment (3, 4). Besides, drug resistance, commonly caused by a loss of the tumor cell's ability to enter apoptosis, is the main cause for the reduction of effectiveness of anti-tumor therapy. Based on the aforementioned details, a large number of agents directly or indirectly modulating apoptosis process are applied to cervical cancer therapy (5).

In this review, we organized novel chemotherapy drugs ongoing clinical trials and expounded their treatment prospects. We also classified new agents in pre-clinical stages and their anti-tumor mechanisms by regulation of the apoptosis process. To summarize, we stated the development prospects of the new use of old drugs and the combined use of drugs for the treatment of cervical cancer.

## Current Drugs for Cervical Cancer Treatment

Except for early-stage cervical cancer, wherein surgical treatment can help achieve the desired curative result, chemotherapy continues to be the standard of cervical cancer care. The common drugs used in clinical settings are listed in Table 1. For micro-invasive carcinomas (stage IA1) and small-volume macroscopic disease (IB1 and IIA1), conization and hysterectomy can help achieve the desired curative result (6). For locally advanced stage, radiation concurrent with cisplatin-based chemotherapy continues to be the cornerstone of the primary treatment (7). Besides platinum drugs, cell cycle specific drugs including paclitaxel, vincristine, gemcitabine, and 5-fluorouracil have radiosensitization capability or synergize the cytotoxic effects of cisplatin. Systemic drug treatment has little effect when used for advanced stage disease. Apart from the standard treatment regimens involving cisplatin and paclitaxel, addition of some emerging drugs can increase the effects of radiation, chemotherapy, and hormone therapy. Those drugs are usually based on molecular targeting. For example, in patients with programmed death ligand 1 (PDL-1)-positive advanced cervical cancer, pembrolizumab, a highly selective IgG4-kappa humanized monoclonal antibody against programmed cell death protein 1 (PD-1) receptor, demonstrated antitumor activity and exhibited a safety profile consistent with that seen in other tumor types (8).

**Table 1:** Common drugs used for cervical cancer treatment in clinical.

		**Drugs**
Cell cycle non-specific drugs		Cisplatin, Carboplatin, Oxaliplatin
Cell cycle specific drugs		Paclitaxel, Vincristine, Gemcitabine, 5-fluorouracil
Targeted therapy	Target VEGF	Bevacizumab, Sildenib, Sunitinib, Pazopanib
	Target EGFR	Cetuximab, Lapatinib, Gefitinib, Erlotinib
	Block Signal Transduction	CCI-779, Gendicine
	Target PD-L1	Pembrolizumab

## Emerging Drugs Ongoing Clinical Trials

### Immune Checkpoints

Considering drug resistance and the low survival rate of advanced stage disease, emerging agents like pembrolizumab are springing up (Table 2). Due to the researchers' effort for the discovery of targetable molecular alterations in cancer, targeted therapy provides a new strategy for cervical cancer treatment. Immune checkpoint is a kind of co-stimulatory and inhibitory signal for regulating the antigen recognition of T cell receptor (TCR) in the process of immune response (9). After Merck & Co firstly developed pembrolizumab and it was approved for the treatment of metastatic malignant melanoma (10), more checkpoint inhibitors were found and explored for its clinical anti-tumor effect. Durvalumab, exerts an antitumor effect by blocking the binding of PD-L1 to PD-1. Durvalumab has entered phase III clinical trials to determine its efficacy and safety in combination with platinum-based chemoradiotherapy in cervical cancer patients (NCT03830866) (11). Besides, other drugs targeted PD1, like Nivolumab (NCT02257528), Atezolizumab (NCT03340376), and Cemiplimab (NCT03257267), were also synthesized and have completed phase II or phase III clinical trial periods.

**Table 2:** Emerging drugs ongoing clinical trials for cervical cancer treatment.

**Classification**	**Drug**	**Study**	**Phase**	**Mechanism**
Immunotherapy	ADXS11-001	NCT02853604	III	Stimulate adaptive immune system resulting in activated CD4+ and CD8+; alters the tumor microenvironment
	Durvalumab	NCT03830866	III	Block the binding of PD-L1 to PD-1
	AGEN2034	NCT03104699	I	Block PD-1 from interacting with its ligands PD-L1 and PD-L2
	Nivolumab	NCT02257528	II	Target PD-1
	Atezolizumab	NCT03340376	II	Blocking the PD-L1/PD-1 immune checkpoint
	Daratumumab	NCT02488759	II	Anti-CD38 antibody
	Cemiplimab	NCT03257267	III	Target PD-1
	GX-188E	NCT03444376	II	DNA vaccination
Target other molecules	Triapine	NCT02466971	III	Inhibition of RNR results in the depletion of dNTP precursors of DNA
	Nelfinavir	NCT03256916	III	Several mechanisms including autophagy disruption, apoptosis, induction of ER stress, and the inhibition of several molecules or their signaling
	Anlotinib	NCT02558348	I	Multi-target tyrosine kinase inhibitor that was designed to primarily inhibit VEGFR2/3, FGFR1-4, PDGFR α/β, c-Kit, and Ret
	Tisotumab vedotin	NCT03438396	II	An antibody-drug conjugate directed against tissue factor (TF) and linked with the cytotoxic drug monomethyl auristatin E
	Mapatumumab	NCT01088347	I-II	Targets and activates the tumor necrosis factor, apoptosis-inducing ligand receptor-1 (TRAIL-R1)

### Targeting Tissue-Specific Proteins and Tyrosine Kinase Inhibitor

Oncoproteins and kinase inhibitors have been studied for their anti-tumor activity for a long time. For example, tisotumab vedotin was designed to conjugate directly against tissue factor and exert its anti-tumor activity (12). An undergoing phase II study (NCT03438396) intends to explore the efficacy and toxicity of tisotumab vedotin in combination with bevacizumab treatment. Anlotinib is a novel multi-target tyrosine kinase inhibitor playing its anti-tumor effect by inhibiting VEGFR2/3, FGFR1-4, PDGFR α/β, c-Kit, and Ret (13). This inhibitor was approved by the China National Medical Products Administration (NMPA) for the treatment of patients with advanced non-small cell lung cancer (NSCLC). A phase I-II clinical trial (NCT02558348) is being conducted to evaluate the curative effect of anlotinib on cervical cancer. Recurrent and metastatic cervical cancer are medical conditions that are difficult to treat, however, a latest research study announced that a phase II trial (NCT03816553) based on 55-Apatinib plus camrelizumab in patients with advanced cervical cancer exhibited a nearly 60% objective response rate (ORR). This is a significant achievement when compared with various medications in the past with a 30% ORR (14). Although emerging target therapy and immunity therapy have brought promising prospects for the treatment of many tumor types, the progress of targeted therapy for cervical cancer is particularly slow due to its unclear pathogenesis. Thus, further efforts should be made to find a new direction for the treatment of cervical cancer.

### Inhibitor of Apoptosis Proteins (IAPs)

As apoptosis process matters tumorigenesis progression, SMAC mimetics, designed according to the endogenous IAPs antagonist second mitochondria-derived activator of caspases (SMAC), and IAP inhibitors, represent important classes of novel agents currently in phase II/III clinical trials. Nelfinavir, a protease inhibitor of human immunodeficiency virus (HIV), can induce endoplasmic reticulum (ER) stress and promote apoptosis through inhibition of several molecules or their signaling such as Akt, NF-kB, matrix metalloproteases, and proteasome. An undergoing phase III clinical trial (NCT03256916) intends to explore the safety and dosing of nelfinavir (15). Mapatumumab is another agent that exerts anti-tumor activity by targeting and activating the tumor necrosis factor and apoptosis-inducing ligand receptor-1 (16).

As drugs directed at inducing tumor-cell apoptosis are regarded as important treatment modalities, apoptotic signaling pathway inhibitors are suggested to be promising for the individualized treatment of tumors. PI3K/Akt, MAPK, NF-κB, and JAK/STAT3 are four classical apoptosis signaling pathways of cervical cancer. Jingjing Liu revealed that PI3K inhibitor (BYL-719/ LY294002) suppressed tumor migration and invasion by targeting β-catenin and matrix metalloproteinase-2/9 and overcame paclitaxel-mediated resistance in cervical cancer (17). However, clinical trials involving PI3K inhibitor (BYL-719/ LY294002) tested its efficacy for use in treatment regimens for head and neck cancer or breast cancer (NCT02051751). BIRB796, a novel p38/MAPK inhibitor, has recently been proven to enhance the antitumor effects of VX680 in cervical cancer (18). 9AA, a compound screened for activating p53, that inhibits NF-κB is a promising drug for cancer treatment (19). AG490, an JAKs inhibitor that has been widely used in recent decades, inhibits the growth and invasion of tumor cells by competing with receptor tyrosine kinase for binding sites (20).

Although an increasing number of inhibitors have demonstrated anti-tumor properties, inhibitors are not allowed to enter the clinical trial stage due to limitations including unfavorable pharmacokinetic properties and high toxicity. On the other hand, because of the complex signal transduction pathways among cells, even if some signal pathways of tumor cells are suppressed, others can still produce compensatory effects on the treatment. Hence, the anti-tumor strategy that inhibits signal transduction should also be a combination of multiple pathways and multiple targets, which may help achieve better results.

## Novel Agents for Cervical Cancer Treatment at Preclinical Stage

Due to the emergence of drug resistance in the conventional treatment of cervical cancer, uncertainty concerning the efficacy and safety of emerging drugs, and other disadvantages associated with both current drugs and emerging agents (Table 3), there is need for superior novel agents. Here we classified novel agents at a preclinical stage to be used for cervical cancer treatment and summarized their underlying anti-tumor mechanisms.

**Table 3:** Main advantages and disadvantages of current drugs and novel agents.

	**Advantages**	**Disadvantages**
•Current drugs	•Wide applicable •Rapid onset	•High toxic and effective •Killing normal cells •Resistance
•Novel agents	•High precision •Hypotoxicity; Slight side effect •Lasting effect and prolong the survival time •No hospitalization	•Only applicable for patients with specific gene mutations •Expensive •Resistance •Some of them lack complete clinical trial data

### Nature Products From Plants

**Figure 1:**
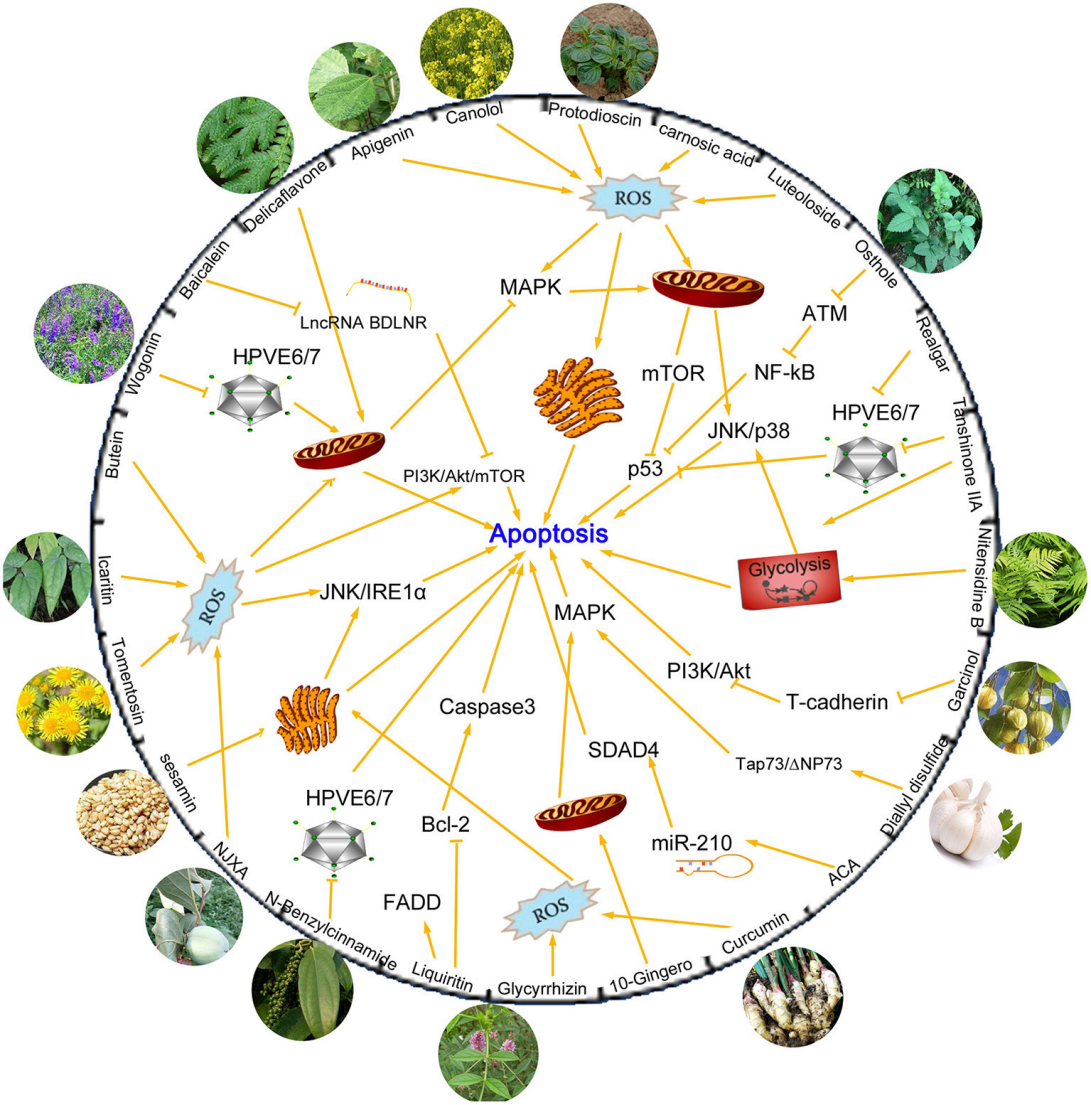
Chemical compounds isolated from plants and mechanisms of their anti-tumor activities by inhibiting apoptosis process. Many compounds are found to exert significant anti-tumor activity. Some of these compounds kill cancer cells by increasing production of ROS levels and then activate mitochondrial or endoplasmic reticulum apoptosis related pathways. HPVE6/E7 proteins are other targets suppressed by novel compounds, which can effectively inhibit the proliferation of cervical cancer cells. MAPK, PI3K/AKT/mTOR, JNK/p38, p53, NF-κB are classical apoptotic pathways and are involved in the regulatory network of drugs that induce apoptosis.

Natural products isolated from medicinal or edible plants are usually very small molecules, but have been proven to possess interesting biological activity, including anti-tumor effect (21). Traditional Chinese medicine has been known as a critical resource for anti-cancer application. Chemical compounds isolated from plants have been explored for their potential anti-tumor activity due to its steady supply, long-lasting curative effects, and mild complications (Figure 1).

Carnosic acid and Luteoloside are two kinds of active substances isolated from Chinese herbs that exert an antioxidant effect. It has been described in literature that carnosic acid and luteoloside can be used as antitumor drug agents for cervical cancer treatment. The agents exert their effect by induction of apoptosis via modulation of reactive oxygen species (ROS) and mitochondrial signaling pathways by activating downstream molecules like mTOR and p53 (22, 23). Tanshinone IIA and Realgar are agents found in Danshen and Xionghuang that exert excellent anti-tumor activity. They possess significant anti-viral activity by repressing HPV oncogenes and inducing apoptosis through a p53-dependent pathway, making them potent therapeutic agents for the prevention and treatment of cervical and other HPV-related cancers (24, 25). Besides, Tanshinone IIA was also reported to induce apoptosis of cervical cancer cells via inhibition of glucose metabolism (26). Osthole (7-methoxy-8-isopentenoxycoumarin), a monomer compound that is extracted from Cnidiummonnieri (L.) Cusson, has been shown to enhance antitumor activity and irradiation sensitivity of cervical cancer cells by suppressing ATM/NF-κB signaling (27).

Apart from medical plants, active substances could also be found in edible plants, such as ginger, garlic, pepper, sesame, rapeseed oil, garcinia, and other seasoning ingredients plants. Curcumin, 10-Gingero, and 1'S-1'-Acetoxychavicol Acetate (ACA) are three ginger-derived natural compounds that possess anti-cancer potential. Curcumin promotes ER stress-mediated apoptosis in cervical cancer cells through increase of cell type-specific ROS generation (28). 10-Gingero activated apoptosis indicators in mitochondria dependent pathway, leading to PI3K/AKT inhibition and AMPK activation and inducing mTOR mediated cell apoptosis in Hela cells (29). MicroRNAs (miRNAs) are short non-coding RNAs that regulate genes post-transcriptionally and are involved in regulation of several biological process including cell death (30). Phuah reported that ACA could inhibit cell proliferation and promote apoptosis by down-regulating miR-210 expression and down-regulation of miR-210 conferred sensitivity toward ACA in cervical cancer cells by targeting SMAD4 (31). N-Benzylcinnamide, a natural compound derived from *Piper submultinerve*, induced apoptosis in HPV16 and HPV18 cervical cancer cells via suppression of E6 and E7 protein expression (32). Protodioscin is the main steroidal saponin of the Tribulus and Dioscoreae families, which was reported to induce apoptosis through ROS-mediated endoplasmic reticulum stress via the JNK/p38 activation pathways in human cervical cancer cells (33).

### Synthetically Modified Drugs

Many kinds of phytochemical substances are considered new strategies for cervical cancer treatments. Among them, plant polyphenols, a kind of secondary metabolite with polyphenol structure widely existing in plants, display potent anti-tumor activity via regulation of cell proliferation, tumor growth, angiogenesis, metastasis, inflammation, and apoptosis (34). Resveratrol, flavonoids, and anthocyanin are polyphenolic phytoalexins that have been proven to have an effect on anti-tumorigenesis (35), however, the poor bioavailability of these compounds has been highlighted as a critical issue to be solved before they are clinically used. To overcome this limitation, the synthesis of plant polyphenols modified for structural optimization with higher activity has become a topic of interest globally. Flavonoid analogs (WYC02-9) (36), curcumin analogs (MS17) (37), resveratrol analogs (MPDB) (38), anthocyanin analogs (idaein chloride) (39), and other active molecular derivatives were obtained and their anti-tumor activity were investigated. When anti-tumor agents exert their action, apoptosis is triggered. Researches showed that those biomolecular derivatives activated apoptosis via both intrinsic (mitochondrial) and extrinsic (Fas receptor) pathways by inducing G2/M phase cell cycle arrest and downregulating E6 and E7 oncogene expression in cervical cancer cells (40, 41).

Along with phytochemical molecules, the synthesis of biologically active organic compounds is another direction for synthetic drug development. Novel biphenylurea compounds (TPD7) (42), piperazine derivatives (1-Phenylpiperazine clubbed with 2-azetidione derivatives) (43), and quinazoline (CP-31398) (44) are reported to be promising leads for the development of effective antitumor agents. (1S, 4S)-2,5-diazabicyclo heptane system was found to be superior than the piperazine counterpart 11, which could inhibit cancer cell proliferation by inducing apoptosis through caspase-3 activation (45). A novel series of triphenylstannyl 4-((arylimino) methyl) benzoates (2–8) exhibited enhanced cytotoxic efficacy compared to cisplatin by inducing apoptotic cell death, attributable to the tin-assisted generation of reactive oxygen species (40).

### Specific Protein Inhibitor

The malignant progression of cancer cells is often related to a variety of molecular abnormalities, thus targeting these molecules is a new direction for development of new drugs. Oncoprotein inhibitors and protease inhibitors are two main applications of specific protein targeting therapy.

Many kinds of oncoproteins were found to be upregulated in cancer tissues, and inhibition of them could slow down the progression of tumors and improve the survival time of patients. Heat shock protein 90 (Hsp90) plays an important role in mediating cellular homeostasis and stabilizing proteins related to intracellular signal transduction. It is reported to upregulation in various kinds of cancers. Hsp90 Inhibitor, SNX-2112, has been shown to enhance tumor necrosis factor ligand superfamily member 10 (TRAIL)-induced apoptosis of human cervical cancer cells via the ROS-mediated JNK-p53-autophagy-DR5 pathway (46), providing a novel strategy for the treatment of cervical cancer by overcoming cellular mechanisms of apoptosis resistance. Another member of heat shock protein 70 family, mortalin, has been reported to play a key role in many biological processes including cell proliferation, migration, angiogenesis, and apoptosis. Mortaparib as a first dual inhibitor of mortalin and poly (ADP-ribose) polymerase family, member 1 (PARP1), has *in vitro* and *in vivo* tumor suppressor activity, making it a potential anticancer drug (47). Besides, snake venom toxin (NF-κB inhibitor) (48), TFO1 (HMGA1 inhibitor) (49), C646 (elective p300 inhibitor) (50), and GW627368X (a highly selective competitive EP4 antagonist) (51) were also reported to have anti-tumor activity and are promising therapeutic agents.

The application of protease inhibitors can reduce the invasion and metastasis of tumor cells caused by protease hydrolysis to a certain extent, and its inhibitory effect has different degrees of specificity, which can slow down the progress of malignant tumor development (52). There are endless opportunities for research and development of protease inhibitors. In recent years, carbonic anhydrase IX (CA IX) has become the focus of attention for its overexpression in many solid tumor types. CA IX inhibitors, aromatic sulphonamide S-1 and sulphonamides, are potential drug candidates for cervical cancers, that exhibit high effectiveness for cancer cell death by inducing apoptosis (53). Vosaroxin is a quinolone-derivative anticancer agent with inhibitory activity on type II DNA topoisomerases (TOP2). It was reported to induce mitochondrial dysfunction and apoptosis in cervical cancer HeLa cells with involvement of AMPK/Sirt3/HIF-1 pathway (54). Another topoisomerase inhibitor, topotecan, combined with the use of histone deacetylase inhibitor (panobinostat) provoked strong cell death responses in cervical cancer-derived cells via induction of the intrinsic apoptotic pathway (55).

### Bioactive Metabolites Derived From Microorganisms

As mentioned above, natural products and bioactive compounds have gained considerable attention among the newly developed chemotherapeutic agents for their well-defined pharmacokinetic properties. Most of therapeutic natural products are extracted from plant sources. However, the increasing destruction of the host plants, which limits the drug supply required to suffice the steadily increasing pharmaceutical demand, prompts researchers to look for other alternative sources.

Microorganisms, with large numbers and wide distribution, have abundant secondary metabolites. As antibiotics and other microbial-derived medicines extensively benefit human health, microbial products are still important resources for people to find novel drug precursors. A large number of studies have shown that the secondary metabolites of many microorganisms have anticancer activity, mainly actinomycetes, fungi, and bacteria (Table 4). For example, extract of *Penicillium sclerotiorum*, an endophytic fungus isolated from Cassia fistula L. was proven to induce cell cycle arrest, further leading to apoptosis through mitochondrial membrane depolarization in human cervical cancer cells (57). Gliotoxin isolated from marine fungus aspergillus sp. and polysaccharides produced by enterobacter cloacae are also reported to induce apoptosis of cervical cancer cells through an intrinsic apoptotic pathway (60). A mTOR inhibitor in an active fraction of the ethyl acetate extract of Streptomyces sp OA293 was identified by Dan et al. (58) which was proven to promote Bax mediated intrinsic apoptosis and autophagy by involving inhibition of mTOR pathway in cervical cancer cell lines. Rosoloactone, a natural diterpenoid isolated from the endophytic fungus *Trichothecium roseum*, displayed significant antitumor activity *in vitro* by inducing apoptosis in human cervical cancer cells through endoplasmic reticulum stress and mitochondrial damage (56).

**Table 4:** Characteristics of the secondary metabolites derived from microorganisms used for cervical cancer treatment.

	**Origin**	**Anti-tumor mechanism**	**Reference**
Rosoloactone	Trichothecium roseum	Rosoloactone mediates pro-apoptotic effects via the activation of ERS-associated apoptosis and the mitochondria-mediated apoptotic pathway.	(56)
PSE	Penicillium sclerotiorum	PSE leads to activation of mitochondrial pathway of apoptosis and exhibites both antioxidant and anti-angiogenic properties.	(57)
Streptomyces sp metabolite(s)	Streptomyces sp OA293	A mTORC1 inhibitor suppresses activation of both of its downstream targets, 4E-BP1 and P70S6k and Akt expression	(58)
Polysaccharides	Enterobacter cloacae	Induce the apoptosis of cervical cancer cells through an intrinsic apoptotic pathway	(59)
Gliotoxin	Aspergillus sp	Induce apoptosis in Hela and SW1353 cells via the mitochondrial pathway	(60)

### Other Strategies for Cervical Cancer Treatment

Apart from agents mentioned above, there are other kinds of active molecules derived from the body itself, which were proven to have anti-tumor activity and are regarded as promising therapeutic agents. Spermidine, a natural polyamine detected in all eukaryotic organisms, exhibits functions that promote longevity in multiple model systems and may constitute a promising agent for cancer treatment (61). Nicotinamide, a necessary nutrient that is provided by dietary source and supplements, was reported to restrain HeLa cell proliferation and significantly increase ROS accumulation and depletion of L-Glutathione (GSH) at relatively high concentrations (62). Kallistatin, recognized as an endogenous angiogenesis inhibitor, exerts pleiotropic effects in inhibiting tumor growth, migration, apoptosis, and inflammation (63), making it a novel therapeutic target for cervical cancer treatment. Besides, continuous HPV infection is considered the main cause of cervical cancer, leading HPV oncoprotein E6/E7 to be an interesting target in cancer therapies. Oei (64) proposed a kind of hyperthermia therapy that could improve patient outcomes. Hyperthermia therapy mechanism involves clinically relevant hyperthermia temperature of 42°C for 1 h, which could result in E6 degradation, thereby preventing the formation of the E6–p53 complex, and enabling p53-dependent apoptosis and G2-phase arrest.

## News Uses of Conventional Drugs and Drug Combinations

With the rapid development of scientific research, various new medicines have sprung up and there is constant development in the field, which can be a promising development for patients. However, there is still a lack of in-depth understanding and comprehensive knowledge of new agents, including concerns on their usage and adverse reactions. As a result, there are often some deviations in the clinical use, such as the decline of drug efficacy or the appearance of undesirable side effects. Therefore, the popularization and use of a new drug requires a large number of clinical trials to verify the safety and effectiveness, which will require a significant amount of labor, material, and financial resources. Under this limitation, people start to pay attention to conventional drugs for their new uses or their use in combination medicine.

Drug reuse is based on previous research and development with detailed drug formulations and safety information, which means that such drugs can enter clinical trials faster than brand-new drugs. Based on this, new use of old drugs will have broad application prospects. Propofol is an intravenous anesthetic agent used for induction and maintenance of general anesthesia. Apart from the multiple anesthetic advantages of propofol, it exerts a number of non-anesthetic effects including an anti-tumor effect. Many studies have demonstrated that propofol can act as an anti-cancer agent in various types of tumors, such as breast cancer (65), lung cancer (66), and other cancer diseases. In cervical cancer, propofol can promote cell apoptosis via inhibiting HOTAIR mediated mTOR pathway (67). Li et al. also found that propofol could enhance the cisplatin-induced apoptosis on cervical cancer cells *via* EGFR/JAK2/STAT3 pathway (68). Zoledronic acid is often used to treat and prevent multiple forms of osteoporosis, hypercalcemia of malignancy, multiple myeloma, bone metastases from solid tumors, and Paget's disease of bone. Zoledronic acid has been proven to modulate the tumor microenvironment through regulation of angiogenesis and immunity, and is considered a promising anti-cancer agent (69).

**Figure 2:**
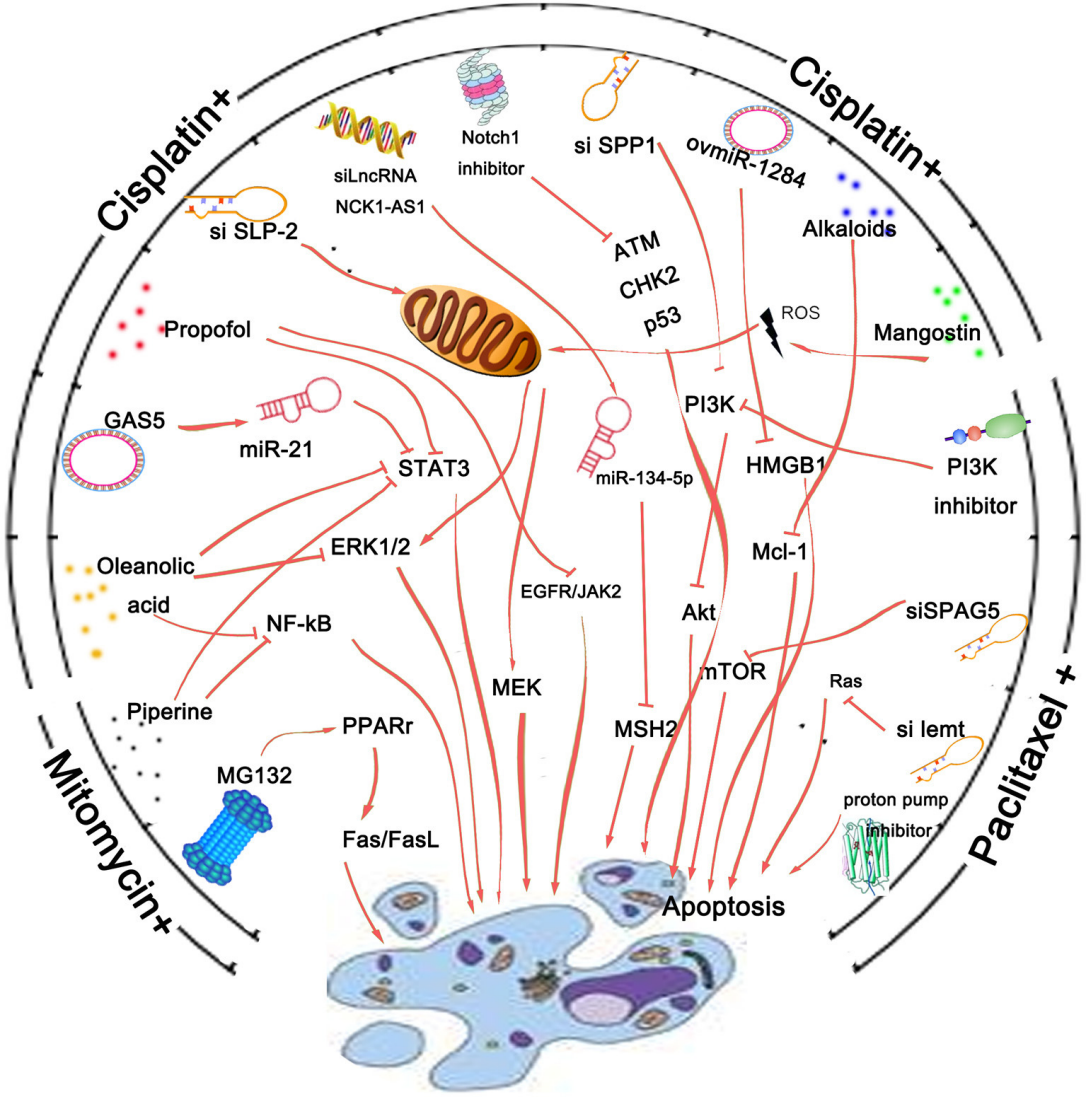
Agents that could overcome drug resistance and increase the sensitivity of chemotherapy drugs by promoting cancer cell apoptosis. Cisplatin, mitomycin, and paclitaxel are three kinds of drugs commonly used clinically. Gene intervention is a pivotal technology used for cancer research. Regulation of crucial oncogene's expression is reported to be involved in apoptotic process of cancer cells and thus enhance the sensitivity of chemotherapy drugs. Besides, inhibitors of key pathway molecules like Notch1 or PI3K are also reported to promote the anticancer effect of cervical cancer chemotherapy drugs. Many agents like substances derived from plants and protease inhibitors or proton pump inhibitor are found to possess a remarkable sensitization effect on cervical cancer cells to paclitaxel, cisplatin, or other conventional drugs by affecting the apoptotic process of cancer cells.

Recently gene intervention technology has provided an effective means for gene therapy of malignant tumors. To achieve the purpose of effective disease treatment, specific methods are used to suppress the expression of a gene or destroy the structure of a gene so that the gene cannot be expressed. With the expansion and progress of research technology, in addition to oncogenes, some microRNAs, noncoding RNAs, and circular RNAs have also become the targets of genetic intervention for their biological activity. Many studies have suggested that gene intervention could help overcome drug resistance, and increase the sensitivity of chemotherapy drugs, or reduce the toxic and side effects of drugs (70, 71) (Figure 2). For example, suppression of long noncoding RNA NCK1-AS1 was reported to increase chemosensitivity to cisplatin by promoting cisplatin-induced apoptosis in cervical cancer (72). Overexpression of growth arrest-specific 5 (GAS5) could attenuate cisplatin-induced apoptosis in cervical cancer by regulating STAT3 signaling via miR-21 (73). These findings make NCK1-AS1 and GAS5 novel targets for improving the chemotherapeutic response and survival of cervical cancer patients.

In addition to gene intervention, many agents like substances derived from plants, protease inhibitors, and other classes detailed above possess a remarkable sensitization effect on treatment of cervical cancer cells with paclitaxel, cisplatin, or other conventional drugs (Figure 2). Despite synergistically enhancing the effect of paclitaxel against paclitaxel-resistant cervical cancer cells, alkaloids from *Piper nigrum* could also enhance mitomycin-C (MMC) therapy of human cervical cancer through suppression of Bcl-2 signaling pathway via inactivation of STAT3/NF-κB (74). α-Mangostin could not only attenuate stemness and enhance cisplatin-induced cell death in cervical cancer stem-like cells, but also prevent nephrotoxicity in BALB/c (nu/nu) mice (75). Inaccessibility of drugs to poorly vascularized strata of tumor is one of the limiting factors in cancer therapy. Proteasomal inhibition was reported to sensitize cervical cancer cells to mitomycin C-induced bystander effect through death receptor-mediated apoptotic pathway (76). Besides, a 5-aminopyrazole derivative lead compound (BC-7) was found to increase the cytotoxic effect of cisplatin in a synergistic manner with combination index CI <0.9 accompanied by highly favorable dose reduction indices (77).

## Conclusion and Future Directions

Although the occurrence of cervical cancer can be prevented with the development and popularization of HPV vaccine, treatment for cervical cancer remains rigorous. The treatment regimen is particularly intensive for locally advanced cervical cancer, which accounts for almost 32% of all stages with a 5-year overall survival rates of approximately 40–50% (78), indicating a disappointing treatment status for relapsed or advanced carcinoma. Advances of genomic knowledge and immunotherapy provide new strategies and directions for cancer therapy. Research concerning targeted therapeutic drugs has proven to possess good application prospects. Bevacizumab, pembrolizumab, and other emerging target drugs are approved to be used alone or with other drugs to treat various kinds of tumors including cervical cancer, colorectal cancer, glioblastoma, hepatocellular carcinoma, etc. The development of such targeted drugs is outstanding, leading the use of tumor-specific signaling or specific metabolic pathways to control “target therapy” and are becoming a topic of interest in cancer research hot spot for cancer research. An increasing number of tumor targeted therapy drugs have been developed and have entered the stage of clinical trials, creating promising treatment regimens for cancer treatment.

At present, it is believed that the occurrence and development of tumors are not only the result of uncontrolled cell proliferation and abnormal cell differentiation, but also related to the imbalance of tumor cell apoptosis. Therefore, a new strategy for cancer treatment involves the interventional regulation of tumor cells' apoptosis process. SMAC mimetics and IAP inhibitors are developed and many clinical trials against such antagonists are being carried out to test their toxicity, safety, and dosing characters. Another treatment strategy in connection with apoptosis is the invention of signaling pathway inhibitors. The development and clinical trial results of inhibitors against molecules related to these pathways have been producing good results, making them potential anti-tumor drugs, which can improve patients' survival rate.

Although targeted therapy of tumors shows good clinical application prospects, the development of drug resistance and the expenses involved with a targeted therapy regimen greatly limit their promotion and popularization. Therefore, people are still devoted to researching novel anti-tumor drugs to achieve the optimal equilibrium of efficacy and price. Nature products derived from plants or microorganism are easy to obtain and demonstrate an excellent anti-tumor effect. Natural products derived from traditional Chinese medicine have the characteristics of structure and target diversity, making them an important source of drug development. Since 1940s, people have begun to use small molecule drugs to treat tumors. A series of drugs such as alkylating agents, fluorouracil, methotrexate, and cyclophosphamide are on the market, but most of these molecules have extensive side effects. To focus on the research of natural small molecules and avoid the occurrence of adverse drug reactions, drug developers are committed to processing and modifying small molecule drugs with specific structures through biosynthesis and chemical synthesis methods. The small molecular drugs exert excellent anti-tumor effects on various kinds of tumors; however, low drug utilization is often a troubling problem, which limits the application of new drugs. Hence improving drug utilization has become a key research of drug development.

The continuous innovation of new material application technologies provides unique advantages for drug delivery systems. Drugs carried by nanomaterials have the characteristics of small particles, large specific surface area, high surface reaction activity, many active centers, and strong adsorption capacity. Therefore, nano-medicine can improve the absorption and utilization of the drug, realize efficient target delivery, prolong the half-life of drug consumption, and reduce harmful side effects on normal tissues. The development formula of nano drug particles includes polymer nanoparticles, micelles, liposomes, dendrimers, metal nanoparticles, solid lipid nanoparticles, etc. In 1995, researchers announced the first liposome-based nano-drug, doxorubicin, for the treatment of tumors (79). Afterwards, with continuous advent of new polymer materials, a mass of nanoparticles (nanospheres, nano capsules) have been discovered and have exhibited significant selectivity and efficiency for drug loading and tumor therapy (80–82). This research has inspired new concepts in nanomedicine, and photothermal effects combined with diagnostic imaging or with drugs bring forward new types of combination therapies. Therefore, the continuous optimization of the drug carrier system will provide convenient and favorable conditions for the treatment, prevention, and diagnosis of various diseases.

## Author Contributions

LL and MW: conceptualization. LL and XL: resources. SY: data curation. LL: writing original draft preparation. MW and BW: writing the review and editing. MW: supervision. All authors have read and agreed to the published version of the manuscript.

## Conflict of Interest

The authors declare that the research was conducted in the absence of any commercial or financial relationships that could be construed as a potential conflict of interest.

## Publisher's Note

All claims expressed in this article are solely those of the authors and do not necessarily represent those of their affiliated organizations, or those of the publisher, the editors and the reviewers. Any product that may be evaluated in this article, or claim that may be made by its manufacturer, is not guaranteed or endorsed by the publisher.
